# Novel 3D Co-Culture Model for Epithelial-Stromal Cells Interaction in Prostate Cancer

**DOI:** 10.1371/journal.pone.0075187

**Published:** 2013-09-20

**Authors:** Xiaolan Fang, Sivanandane Sittadjody, Kenneth Gyabaah, Emmanuel C. Opara, Kethandapatti C. Balaji

**Affiliations:** 1 Institute for Regenerative Medicine, Wake Forest University Health Sciences, Winston-Salem, North Carolina, United States of America; 2 Department of Cancer Biology, Comprehensive Cancer Center, Wake Forest University Health Sciences, Winston-Salem, North Carolina, United States of America; 3 Department of Urology, Wake Forest University Health Sciences, Winston-Salem, North Carolina, United States of America; AMS Biotechnology, United Kingdom

## Abstract

Paracrine function is a major mechanism of cell-cell communication within tissue microenvironment in normal development and disease. *In vitro* cell culture models simulating tissue or tumor microenvironment are necessary tools to delineate epithelial-stromal interactions including paracrine function, yet an ideal three-dimensional (3D) tumor model specifically studying paracrine function is currently lacking. In order to fill this void we developed a novel 3D co-culture model in double-layered alginate hydrogel microspheres, incorporating prostate cancer epithelial and stromal cells in separate compartments of the microspheres. The cells remained confined and viable within their respective spheres for over 30 days. As a proof of principle regarding paracrine function of the model, we measured shedded component of E-cadherin (sE-cad) in the conditioned media, a major membrane bound cell adhesive molecule that is highly dysregulated in cancers including prostate cancer. In addition to demonstrating that sE-cad can be reliably quantified in the conditioned media, the time course experiments also demonstrated that the amount of sE-cad is influenced by epithelial-stromal interaction. In conclusion, the study establishes a novel 3D *in vitro* co-culture model that can be used to study cell-cell paracrine interaction.

## Introduction

Several of the *in vitro* cells co-culture models available to study cell-cell interactions use two-dimensional (2D) Petri dishes or plates [1,2,3]. Yet in most living organisms cells are embedded in a three-dimensional (3D) microenvironment, surrounded by other cells and influenced by soluble factors secreted in the extracellular environment. Alternatively sandwich models can be used for multilayer growth of cells, but limitations are obvious, as cells would alter their morphological features, metabolism and gene expression patterns in 2D culture, especially when they are from higher organisms [4,5]. In addition, conventional 2D cell cultures limit cellular communications and transportation of soluble factors, oxygen and nutrients, removal of wastes and cellular metabolism as present in native biological environments [6,7]. Therefore, it is critical to develop *in vitro* model systems that simulate tissue microenvironments to produce reliable and biologically meaningful experimental results.

3D modeling systems simulating tissue microenvironment were developed to address limitations associated with 2D models [8]. While 3D *in vitro* cell culture models overcome several limitations of 2D models, improvement in 3D modeling is necessary to discriminate specific types of cell-cell interaction such as cell-cell direct, autocrine or paracrine functions. Advances in biomaterials and bioengineering techniques allow use of novel materials such as collagen gels, laminin and Matrigel™ in cell culture, develop synthetic extracellular matrix and create a variety of 3D models [5,9,10,11,12,13,14,15]. Among the biomaterials available, alginate hydrogel possesses preferred properties for cell transplantation, drug delivery and tissue engineering. Alginate is a polysaccharide and a biocompatible polymer derived from brown seaweed. By addition of divalent cations such as calcium or barium, alginate polymers can be ionically cross-linked to form a hydrogel. The hydrophilic nature of the alginate scaffolds enables high cell loading that remain viable and functional in culture [16,17,18]. In addition, the production of alginate hydrogel is relatively simple and encapsulation can be achieved under non-stringent conditions. Various cell types including neuronal cells, osteoblasts, chondrocytes, myoblasts, have been encapsulated, cultured and expanded in alginate hydrogels [19,20,21,22,23].

In this study we established a 3D prostate cancer epithelial-stromal interaction in alginate hydrogel microspheres by co-culturing prostate cancer C4-2 cells (stably transfected with Protein Kinase D1 (PKD1) or control vector) and normal prostate stromal cells (WPMY-1 cells) in the same microcapsule, but in separate sub-layers. This system is ideal to study paracrine influence between the two cell types because direct interaction between epithelial and stromal cells is not allowed. As a proof of principle to study paracrine function, we measured shedding of E-cadherin (sE-cad) in soluble media. The sE-cad is an 80 kDa cleaved fragment of E-cadherin, a transmembrane cell adhesive protein that is dysregulated in several cancers including prostate [3,24,25,26]. Elevated sE-cad has been reported in fluids and serum of patients with a variety of cancers and other diseases [25,27,28,29,30] and serum levels have been shown to correlate positively with metastatic prostate cancer and disease recurrence. Thus, sE-cad is suggested to be a novel biomarker for cancer prognosis. We previously described the down regulation of PKD1 in advanced prostate cancer [31], and that PKD1 promotes the E-cadherin shedding through increased matrix metalloproteinases (MMPs) -2 and -9 secretion [24].

## Materials and Methods

### Cell Culture

C4-2 cells stably transfected with pcDNA3.1 vector (vector cells) or PKD1-GFP (PKD1 cells) were developed in our laboratory as previously described [31]. Normal prostate stromal cells (WPMY-1) were obtained from ATCC. Cells were grown in DMEM medium (high glucose) (HyClone, Cat# SH30243.01) with 10% FBS and 1% Antibioltic-antimycotics (HyClone Cat# SV30079.01) in 15-cm sterile culture plate, and incubated at 37°C with 5% CO_2_. When cells reached 80% confluence, media were removed from each plate and cells were washed with sterile PBS three times, treated with trypsin (HyClone, Cat#SH30236.01) for 20 minutes and transferred to sterile centrifuge tubes. They were washed with PBS again, and then resuspended in DMEM medium for encapsulation.

### Fabrication of microcapsules

The stromal, vector and PKD1 cells were encapsulated in alginate hydrogel using a micro-fluidic device with some modifications of encapsulation as described by Tendulkar et al. and Khanna et al. [32,33]. Briefly, first cell type (stromal or vector or PKD1) was mixed with 1.5% (w/v) ultrapure low-viscosity high-mannuronate alginate (LVM) (NovaMatrix, Sandvika Norway) and extruded through the micro-fluidic encapsulation device. The droplets generated were collected in 100 mM calcium chloride solution. After the cross linking of alginate for five minutes in CaCl_2_, the microcapsules were washed with 0.9% NaCl containing 20 mM CaCl_2_. The microcapsules were then incubated with poly-L-Ornithine (PLO) (0.1% w/v) for 20 minutes to create a PLO layer, which serves as a perm selective basement membrane. The PLO-coated microcapsules were then mixed with the second cell type (stromal or vector or PKD1) suspended in 1.5% (w/v) LVM and encapsulated again using the micro-fluidic device in order to obtain multi-layered microcapsules. The microcapsules were washed with 0.9% NaCl containing 20 mM CaCl_2_ and cultured in DMEM containing fetal bovine serum (10% v/v) at 37°C with 5% CO_2_.

### Viability Assay

For viability assessment of encapsulated cells, a few capsules from each transfected group were taken out and transferred to clean 24-well plates, media were aspirated out carefully and cells stained. Single cell type control staining: CFDA SE (Vybrant® CFDA SE Cell Tracer Kit, Invitrogen) reconstituted in serum-free DMEM (SFM, HyClone) (1:400) was added to each well (500 µl/well) and incubated for 15 minutes at 37°C, 5% CO_2_ in the incubator. Then SFM with CFDA SE was replaced by DMEM with 10% FBS and incubated again for another 30 minutes at 37°C, 5% CO_2_ in the incubator. The serum-containing medium was then replaced with 50 µg/ml of propidium iodide (PI) (Life Technologies**/**Invitrogen, Grand Island, NY) and incubated at room temperature for 2 min and the microcapsules were washed 3 times to remove excess PI. The microcapsules were then observed under inverted fluorescence microscope (Zeiss Axiovert 200M) and imaged. The number of live and dead cells was determined qualitatively from the composite image acquired using Image-Pro plus software (version 6.3.1.542). Double cell type staining: To demonstrate the differential compartmentalization of different cell types in the multi-layered micro-capsules, the inner core cells were pre-stained with Cell Tracker green (Invitrogen, cat# C2925) and the outer layer cells were pre-stained with Cell-tracker Orange (Invitrogen), prior to the synthesis of the multi-layered micro-capsules. Before observation, the microcapsules were stained with SYTOX Blue Nucleic Acid Stain (Invitrogen, cat# S11348) for dead cells. The multi-layered micro-capsules were then imaged using the fluorescent microscope (Zeiss Axiovert 200M).

### Enzyme Linked Immunoabsorbent Assay (ELISA)

To evaluate the paracrine functions of encapsulated cells, the levels of sE-cad was measured in the culture media. Each group of microcapsule was cultured in quadruplets and the spent media was collected every alternate day. While collecting the spent media, the media in each well was mixed thoroughly; 1ml (half of the total volume) was taken out from each well and replaced with 1 ml of fresh complete DMEM with 10% FBS and 1% antibiotics. The sample media was stored in a clean Eppendorf tube at -20 °C. For cells growing in 2D tissue culture treated petri dishes, media were collected when the cells reached 90-100% confluence (three-day incubation). The cell number was counted for each cell line for later normalization. The levels of sE-cad were quantified using ELISA kit from R&D systems, Quantikine (human sE-cadherin) as per manufacturers’ instructions.

## Results

### Cell viability maintained for at least a month in microspheres

**Figure 1:**
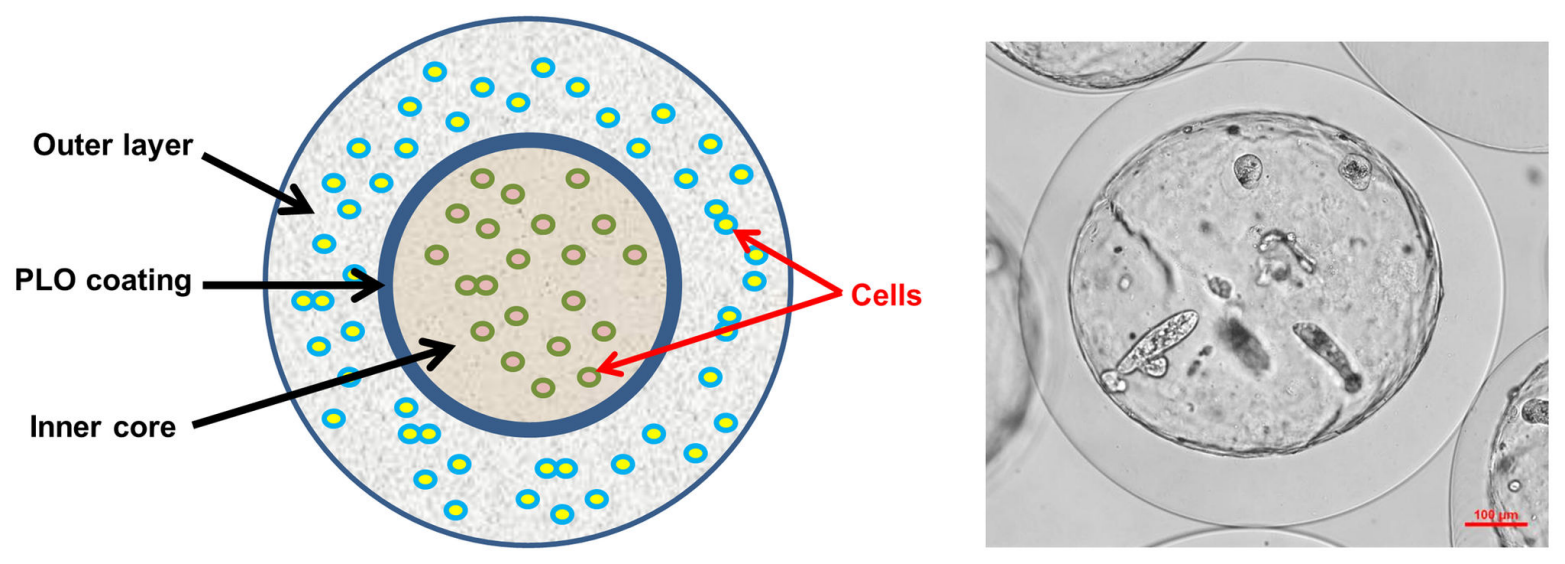
Model of double-layered alginate hydrogel microsphere. Left is a cartoon model of double-layered microsphere. Inner core and outer layer were separated by PLO coating as shown. Cells growing in separate layers were indicated with red arrows. Right is an actual microsphere observed under light microscope. Stromal cells were grown in the inner core for 7 days, and outer layer is blank.

Based on the previous reports of multi-layer microcapsules produced for protein delivery [32,33] we designed double-layer microcapsules which were composed of an alginate inner core and outer layer of alginate (Figure 1) separated by an electrostatically -linked polycationic permselective poly-L-ornithine (PLO) coat with a pore size <150 kDa [32]. Whereas the model prevented direct cell-cell interaction, cellular secretions could permeate through the PLO coat allowing for paracrine activity. As an initial step towards validating the model for paracrine function, we assessed cell viability.

**Figure 2:**
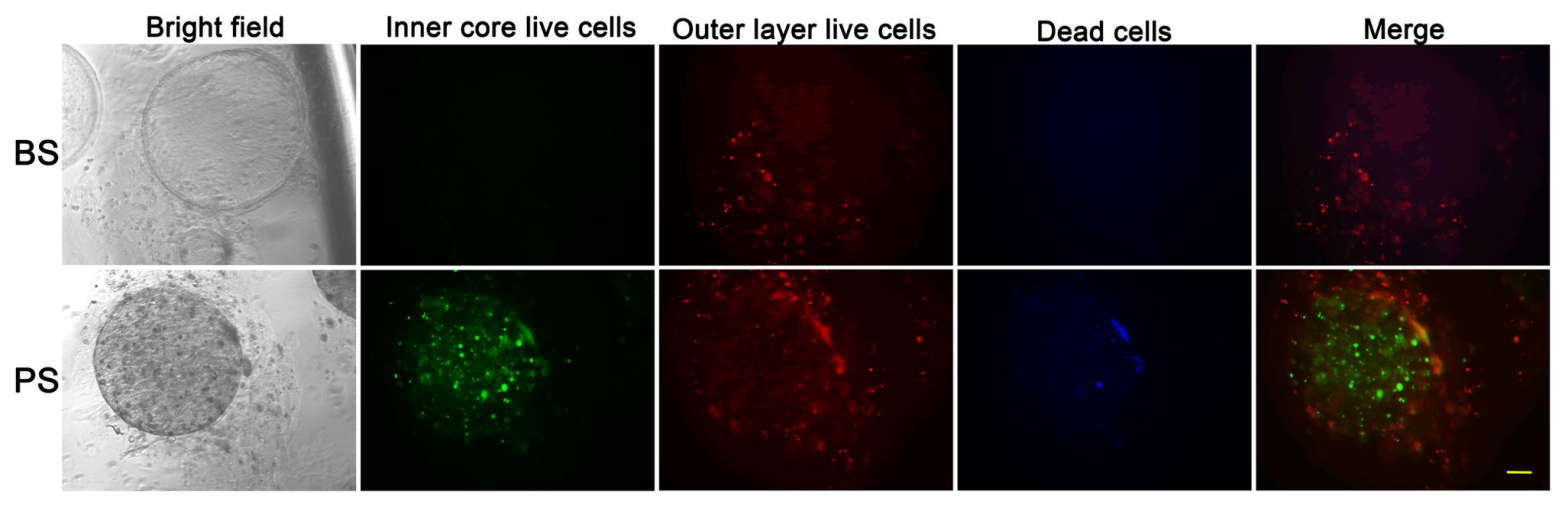
Different lines of prostate cells remained viable in microcapsules for over a month. BS, microcapsules with blank inner core and stromal cells at outer layer. PS, microcapsules with C4-2 PKD1 cells at inner core and stromal cells at outer layer. Green, live cells at inner core. Red, live cells at outer layer. Blue, dead cells. Scale bar, 100µm.

The stromal cells (WPMY-1), C4-2 vector cells (C4-2 cells stably transfected with pcDNA3.1) and C4-2 PKD1 cells (C4-2 cells stably transfected and expressing PKD1) remained viable within inner core or outer layer in the double layered microcapsules for 4 weeks, and remained confined to their respective layers without migration through the PLO (Figure 2). Next, two different cell types were co-cultured in inner core or outer layer of the same microcapsule, and both cell types remained viable in co-culture for at least 4 weeks regardless of the layers or cell type combination (Figure 2). The results demonstrate that alginate microspheres can be used to grow epithelial or stromal cells compartmentalized in different layers *in vitro* for at least a month.

### Cells grown in microspheres demonstrated secretory function

**Figure 3:**
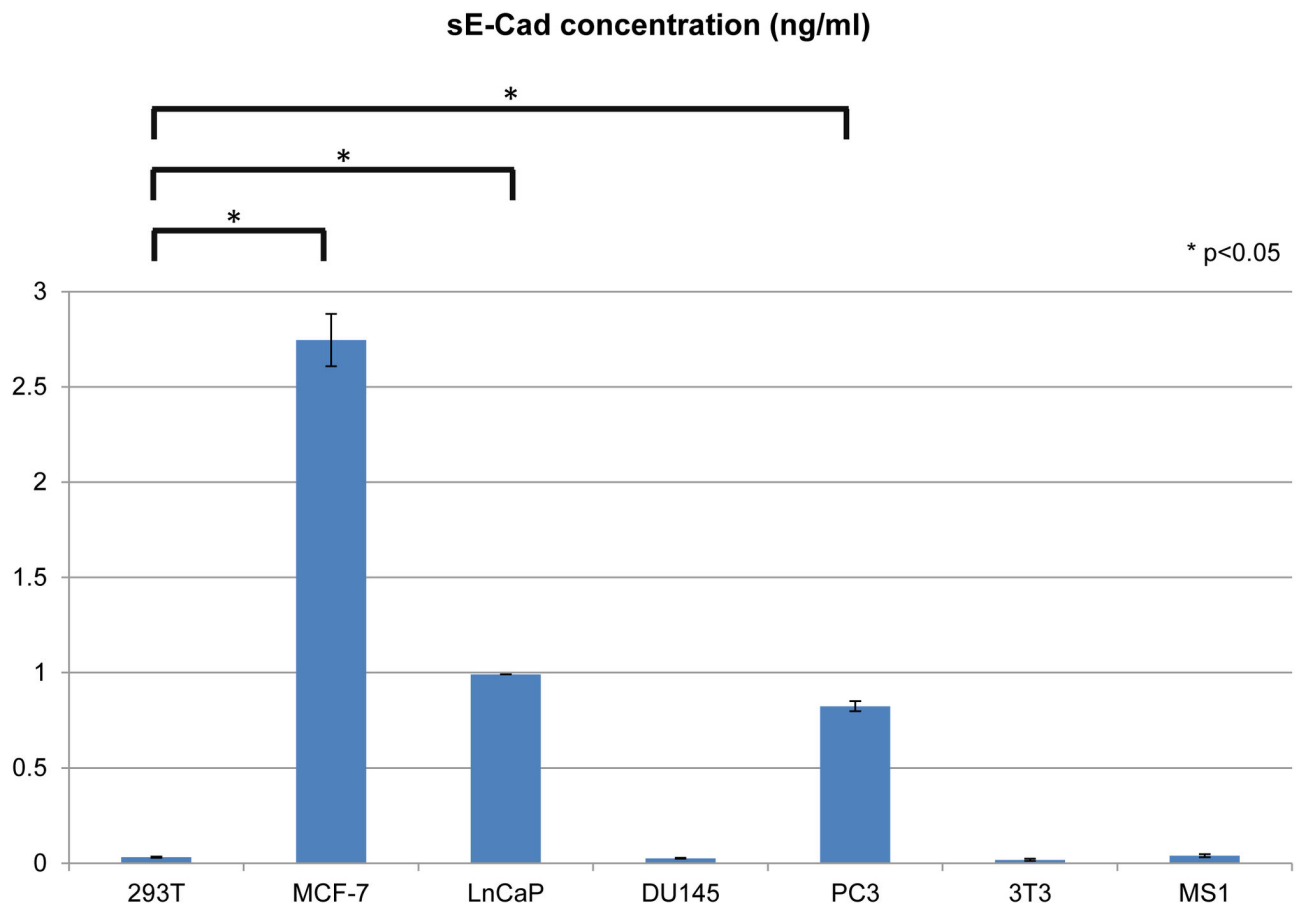
sE-Cad secretions in different human cell types. sE-Cad level was measured by ELISA and was normalized to reflect the secretion of 10^7^ cells for each cell line. sE-Cad level of metastatic cell lines (MCF-7, LnCap and PC3) were compared to HEK293T (as a representative of normal cell lines) and P values were calculated by Student’s t-test. Each column represents the average of three parallel experiments. Error bar represents the standard error.

In order to prove that viable cells in microspheres also remain functional, we measured the concentration of soluble E-cadherin fragment (sE-cad, or shedded E-cadherin) in the conditioned media as a marker of secretory function. E-cadherin is an important transmembrane cell-cell adhesion molecule that is dysregulated in several human cancers [3,34]. The extracellular domain of E-cadherin is cleaved by several extracellular proteases. The cleavage results in shedding of an approximately 80 kDa fragment, which has been shown to be a biomarker of aggressive phenotype in several human cancers including prostate [25,27,28,29,30]. Our previous studies demonstrated that dysregulation of PKD1 influences E-cadherin shedding in prostate cancer cells [24]. Therefore, we used stably transfected C4-2 PKD1 cells to quantify shedded E-cadherin level. In order to include a variety of positive and negative controls, we tested the sE-cad level in conditioned media of different human cell lines, including both normal and neoplastic cells. While some cell types did not show much evidence of E-cad shedding reflected by the low/trace level of sE-Cad, others secreted high levels of sE-cad in the conditioned media (Figure 3). The results showed that metastatic cell lines (MCF-7, PC3 and LnCap cells) have high sE-Cad level, and benign cell lines (HEK293T cells, 3T3 cells and MS1 cells) showed low/trace level of sE-Cad. There were significant differences between sE-Cad levels of metastatic cells and benign cells, which were confirmed by Student’s t-test (Figure 3). These results are consistent with published literature that sE-cad level correlates with cell invasiveness [25].

### Co-culture of epithelial and stromal cells influences shedded E-cadherin secretion as an evidence of paracrine function

**Figure 4:**
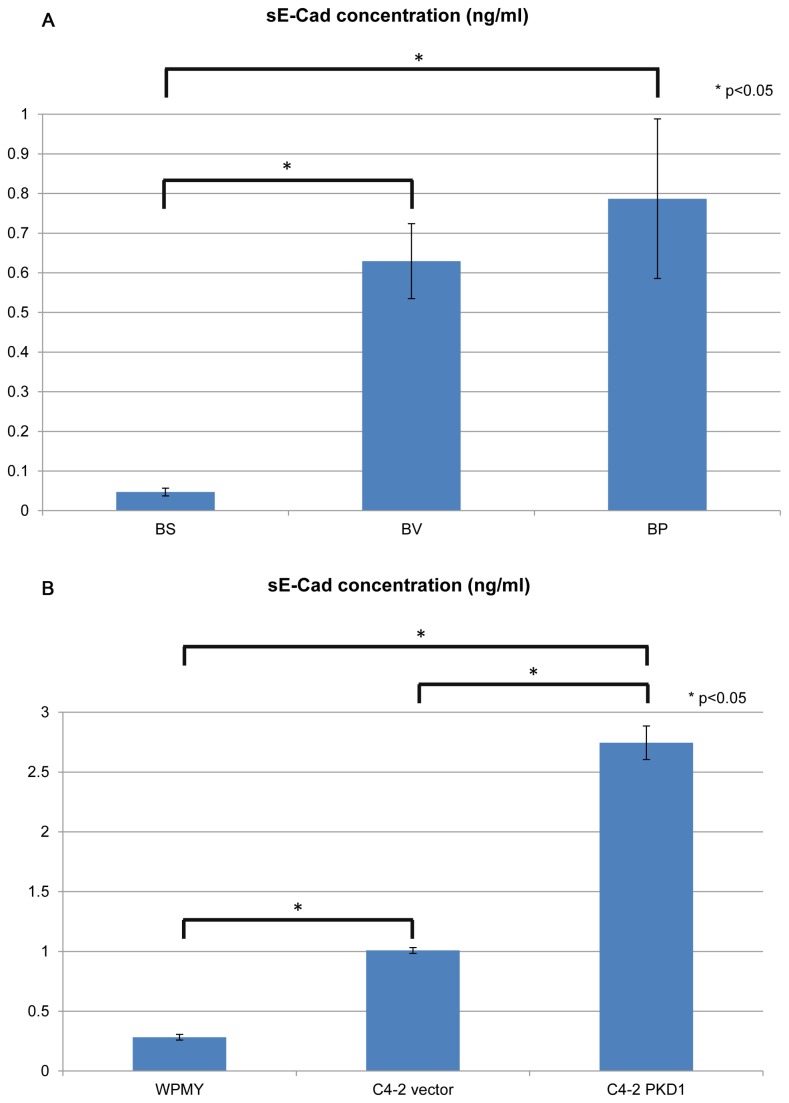
Whereas normal stromal cells do not secret sE-Cad in microcapsules, prostate cancer epithelial cells do. A, cells were cultured in 3D environment for 6 days. BS, microcapsules with blank inner core and stromal cells in outer layer. BV, microcapsules with blank inner core and C4-2 vector cells in outer layer. BP, microcapsules with blank inner core and C4-2 PKD1 cells in outer layer. sE-Cad level was measured by ELISA. B, cells were cultured in 2D environment (petri dish). sE-Cad level was measured by ELISA and normalized to reflect the secretion of 10^7^ cells for each cell line P values were calculated by Student’s t-test. Each column represents the average of three (2D culture) or four (3D culture) parallel experiments. Error bar represents the standard error.

To test paracrine interaction between two different cell lines, stromal cells, C4-2 vector cells and C4-2 PKD1 cells were grown in microcapsules in a variety of combinations and sE-cad in the conditioned media was quantified. When a single cell type was grown in either inner core or outer layer of the microcapsules, stromal cells did not secrete sE-cad, whereas both C4-2 vector cells and C4-2 PKD1 cells did when encapsulated together in separate compartments (Figure 4A). Quantification of sE-Cad was performed for cells cultured in 2D environment and similar results were observed (Figure 4B). The results validate reliability of the model to discriminate epithelial from stromal cells’ functions.

**Figure 5:**
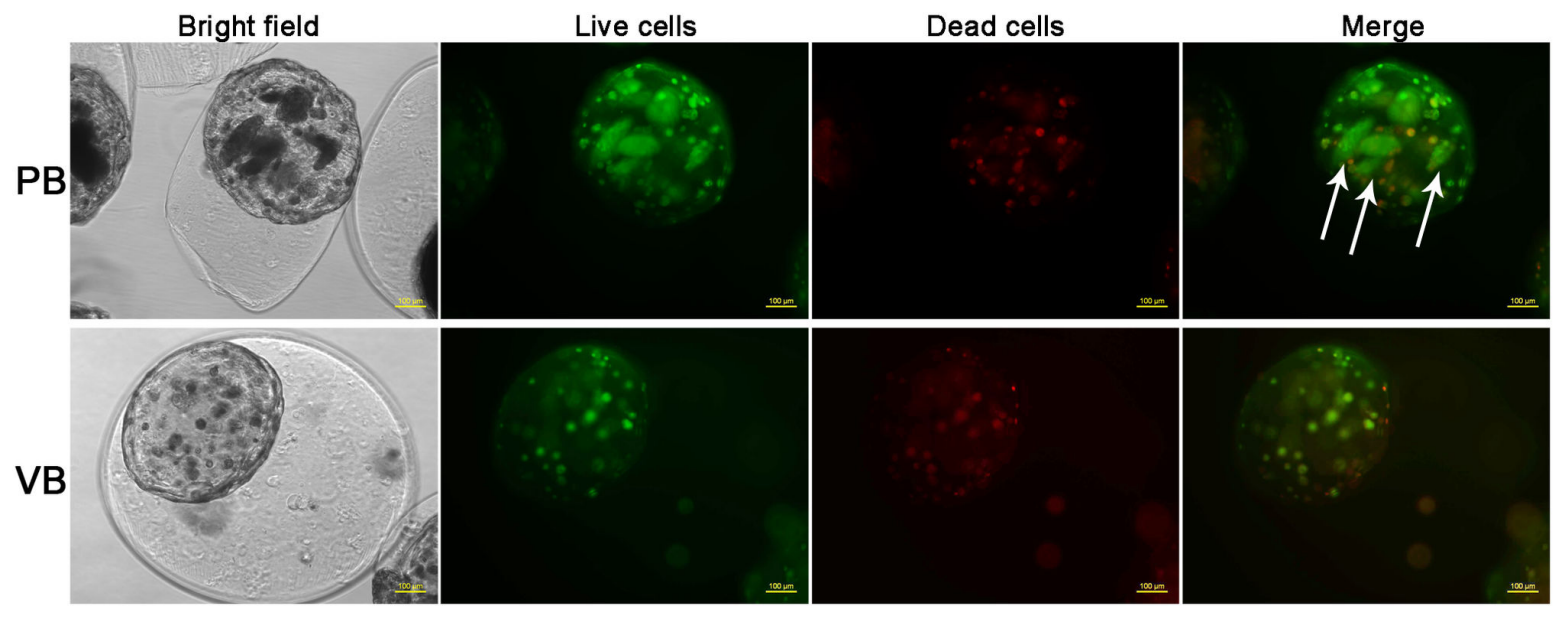
PKD1 expression induced formation of compact cell colonies. Microcapsules incubated for 23 days are shown. PB, microcapsules with C4-2 PKD1 cells at inner core and blank outer layer. VB, microcapsules with C4-2 vector cells at inner core and blank outer layer. Green, CFDA staining for live cells. Red, Propidium iodide (PI) staining for dead cells. Arrows indicate cell colonies. Scale bar, 100µm.

**Figure 6:**
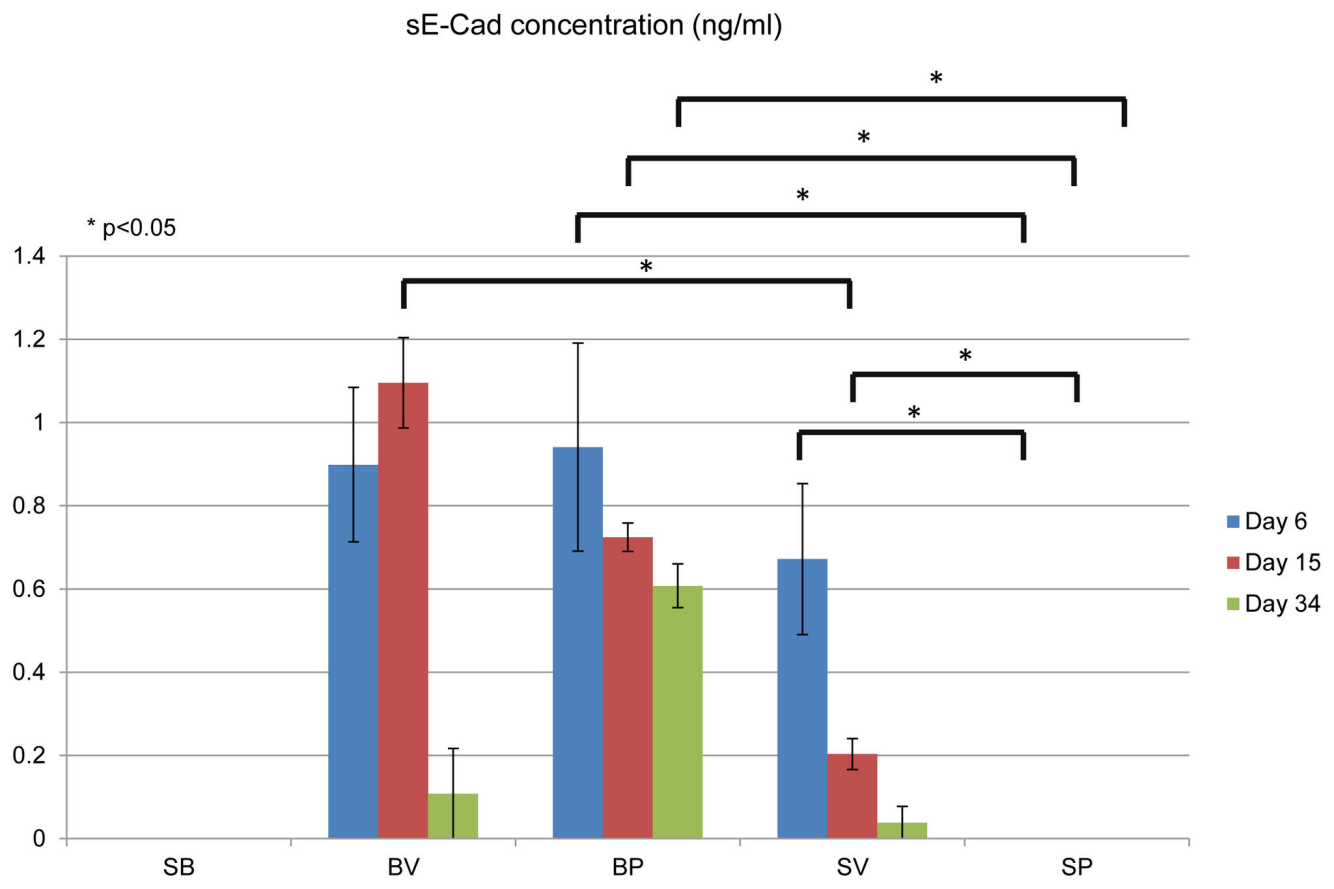
Co-culture of prostate cancer epithelial cells and normal prostate stromal cells decreased sE-cad secretion by the epithelial cells. sE-cad level was measured by ELISA following co-culture for 30 days. SB, microcapsules with stromal cells at inner core and blank outer layer. BV, microcapsules with blank inner core and C4-2 vector cells in outer layer. BP, microcapsules with blank inner core and C4-2 PKD1 cells in outer layer. SV, microcapsules with stromal cells at inner core and C4-2 vector cells in outer layer. SP, microcapsules with stromal cells at inner core and C4-2 PKD1 transfected cells in outer layer. P values were calculated by Student’s t-test. Each column represents the average of four parallel experiments. Error bar represents the standard error.

C4-2 PKD1 cells increased secretion of sE-cad compared to C4-2 vector cells (control) (Figure 4), which is consistent with our prior publications [24]. We have also previously demonstrated that PKD1 up-regulated E-cadherin expression [24]. In the current experimental model C4-2 PKD1 cells but not C4-2 vector cells aggregate to form compact colonies (after cultured for three weeks in microcapsules) (Figure 5), which is a well-established phenotype of increased E-cadherin expression. When epithelial and stromal cells were co-cultured in independent layers within the same microcapsule, the amount of sE-cad secreted by C4-2 cells (both vector and PKD1 transfected cells) was decreased, suggesting stromal cells influence on epithelial sE-cad secretion (Figure 6). Because the stromal and epithelial cells are compartmentalized and unable to interact directly, we postulate that stromal cells influence epithelial cell secretory function via paracrine mechanism.

## Discussion

The double layered microsphere model described in this study is a 3D environment which simulates the *in vivo* microenvironment, amenable to easy and scalable material production, purification and processing, no discernible cytotoxicity, and is chemically compatible with aqueous solutions and physiological conditions. Unlike other currently available 3D *in vitro* model, the microsphere model in this study allows to specifically analyze paracrine interaction between different types of cells. However, this model also exhibits certain limitations. The amount of quantifiable secretion of sE-cad of cells growing in inner core is lower than that of the same number and type of cells grown in outer layer of the microcapsule (data not shown). This decreased secretion may be due to the fact that sE-cad secreted by cells in the inner core needs to diffuse through two layers of hydrogel before entering the conditioned media. It is also possible that the permselectivity of PLO coat decreased the secretion of sE-Cad. To optimize this model for detection of other specific proteins, the chemical features of cross-linked PLO coat may need to be optimized for specific study needs. The influence of exosome should also be taken into consideration, as E-Cadherin was identified in microvesicles purified from normal murine dendritic cells and human cancer cells [35,36]. It remains possible that sE-Cad could be trapped in the exosomes, yet little is known about how microvesicles regulate cell-cell interaction in the paracrine system.

Another concern is the sustained mechanical property of alginate hydrogel, as ionically cross-linked alginates showed decreased gel strength after 90 days *in vitro* [37]. To solve this problem, stable covalent cross-links may be introduced into alginate hydrogels using bi-functional cross-linkers. Also, it is possible that an extended period of *in vitro* incubations may result in an imbalance in the prevailing ion concentrations (which is necessary to maintain microcapsule stability), but not under *in vivo* conditions, as no degradation of these alginate microspheres was observed for at least three months in our recent *in vivo* studies (unpublished data). In spite of these limitations, the model as described can be used effectively to study the paracrine epithelial-stromal interaction.

Epithelial-stromal interactions play a major role in normal development and neoplastic transformation. In normal human prostate the stromal cells are mainly composed of smooth muscle cells and fibroblasts, while the remainder are made of endothelial cells, pericytes, lymphocytes and macrophages [38]. It has been reported that carcinoma-associated stroma (CAS) could change the cell morphology/growth rate/aggressiveness of neoplastic human prostatic epithelial cells, but not normal prostatic epithelial cells [39]. Similar effects were reported using prostate carcinoma cell in co-culture with pleuripotent bone stromal cells [40]. Normal fibroblasts do not demonstrate such transformative effect on epithelial cells, suggesting a characteristic epithelial-stromal interaction during neoplastic process cells [39]. In this study, we demonstrate that one possible mechanism of stromal influence on epithelial cancer cells is through a paracrine mechanism. As a proof of principle we demonstrated reduction in sE-cad secretion by prostate cancer cells in presence of stromal cells and the model could presumably be used to study other cellular functions influenced by paracrine mechanism.

Stromal interaction influences proliferation, apoptosis and metastasis of epithelial cells. In pancreatic cancer, an antagonist of Hedgehog (Hh) inhibited tumor growth only when cancer-associated stroma exists, indicating signaling pathway dependent on stroma [41]. In prostate, it was reported that stromal factors, such as caveolin-1 and thymidine phosphorylase were related with tumor aggressiveness [42]. Other novel proteins that play a role in epithelial-stromal interaction have been identified by transcriptional profiling in prostate [43]. One of them, Decorin, was downregulated in prostate cancer [43]. Decreased Decorin resulted in a significant reduction of E-Cadherin both *in vivo* and *in vitro*, and physical interaction between Decorin and E-Cadherin was confirmed by co-immunoprecipitation [34]. Decorin expression is strictly limited in mesenchymal/stromal cells, but not in epithelial cells in prostate [43], and its expression is significantly decreased in carcinoma-associated stroma [43]. Whether Decorin also plays a role in shedding of E-cadherin remains unknown.

Another important group of extracellular matrix proteins, MMPs, could be possible candidates of stromal regulators that influenced the E-cad shedding. We previously showed that PKD1-induced secretion of MMP-2 and -9 increases E-cadherin shedding and suppresses cell proliferation [24].

In summary, the study demonstrates the feasibility and utility of using 3D alginate microsphere model to study paracrine function of cells. In particular, we used the model to explore epithelial-stromal interaction and demonstrated that normal prostate stromal cells influence secretion of sE-Cad by prostate cancer epithelial cells by paracrine interaction. The model could be used to validate additional cell lines and paracrine interaction of different types of cells.
